# A novel mitochondria‐related core gene signature to predict the prognosis and evaluate tumour microenvironment in CESC single‐cell validation

**DOI:** 10.1111/jcmm.18265

**Published:** 2024-03-27

**Authors:** Lingxiao Ying, Lin Kong, Xiaoxiao Qiu, Aihua Cheng, Qijun Wang, Limeng Xiu, Jinmei Shi, Yanfei Tao, Zhihong Chai

**Affiliations:** ^1^ Department of Gynecology Taizhou Municipal Hospital, Medical College of Taizhou University Taizhou China

**Keywords:** cervical cancer, immunotherapy, mitochondria‐related genes, prognosis, TCGA, tumour microenvironment

## Abstract

Mitochondria and their related genes (MTRGs) are pivotal in the tumour microenvironment (TME) of cervical cancer, influencing prognosis and treatment response. This study developed a prognostic model using MTRGs to predict overall survival (OS) in cervical squamous cell carcinoma and endocervical adenocarcinoma (CESC), aiming for personalized therapy. Analysing 14 MTRGs like ISCU and NDUFA11 through techniques such as univariate Cox regression, we found that a low mitochondrial (MT) score is associated with better survival, while a high MT score predicts poorer outcomes. The TME score, particularly influenced by CD8 T cells, also correlates with prognosis, with a high score indicating favourable outcomes. The interplay between MT and TME subtypes revealed that the best prognosis is seen in patients with a low MT and high TME score. Our findings highlight the role of MTRGs as potential biomarkers and therapeutic targets in cervical cancer, offering a novel approach to improving patient outcomes through a more nuanced understanding of mitochondrial function and immune interactions within the TME. This model presents a promising avenue for enhancing the precision of prognostic assessments in CESC.

## 1 INTRODUCTION

Cervical squamous cell carcinoma and endocervical adenocarcinoma (CESC), accounting for an estimated 570,000 new cases and 311,000 female fatalities annually in 2018, represent a leading cause of mortality in women and rank as the fourth most prevalent malignancy globally.^1^ China and India, with respective populations of 106,000 and 97,000, reported death rates of 48,000 and 60,000, collectively contributing to over one‐third of the total CESC patient count worldwide.^2^ Remarkably, CESC poses a substantial public health challenge, particularly in women residing in resource‐constrained environments. This challenge is linked to persistent high‐risk human papillomavirus (HPV) infection, multiple sexual partners, high parity, long‐term oral contraceptive use and cigarette smoking.^3^, ^4^ Therapeutic strategies for CESC, depending on the disease severity at diagnosis, may include adjuvant or combination therapy for locally advanced disease or surgical treatments in the early stages. Despite surgery, chemotherapy and radiation being standard in high‐income countries, cervical cancer remains the primary cause of death in 42 countries, the majority of which are low‐income and lower‐middle‐income countries (LMICs). Strikingly, despite the existence of early detection methods, effective therapeutics and cervical screening initiatives, CESC is often diagnosed at an advanced stage, resulting in a high mortality rate. The 5‐year survival rates for stages I, II, III and IV are 81%–96%, 65%–87%, 35%–50% and 15%–20%, respectively. Most patients with advanced‐stage CESC succumb to recurrence and medication resistance within 3 years.^5^, ^6^ Predictions of patient survival are currently reliant on the International Federation of Gynaecology and Obstetrics (FIGO) stages. Given the high morbidity, late manifestations and mortality, biomarkers may be necessary for early identification of patients with poor prognosis and for intensifying therapy to improve patient care.

The tumour microenvironment (TME) encompasses a complex network of tumour cells, tumour‐infiltrating immune cells and the stromal compartment, playing a crucial role in forecasting responses to cancer therapies. Mitochondrial dysfunctions and abnormalities in mitochondria‐related genes (MTRGs) are integral to the TME's influence on tumour genesis and progression. Despite the recognised significance of MTRGs in various cancers, their comprehensive impact on CESC remains underexplored. Aberrations in MTRGs, signifying mitochondrial malfunctions, are now seen as key contributors to the altered bioenergetics characteristic of cancerous cells. The metabolic adaptations of mitochondria to the demands of solid tumours lead to changes that can enhance the production of reactive oxygen species (ROS) and eliminate harmful agents, thereby affecting the invasive capabilities of tumour cells. Enhanced heme synthesis, pivotal for the electron transport chain in mitochondrial oxidative phosphorylation, might reduce oxidative metabolism and glycolysis, inhibiting tumour growth.^7^, ^8^

The propensity for gene mutations of moderate penetrance is notably higher in cancer patients, being 2–4 times greater than in the general population.^9^ Recent clinical studies and extensive sequencing projects have highlighted the prevalence of mitochondrial DNA (mtDNA) mutations in tumours and their potential role in cancer development. The aberrant expression of MTRGs during the progression of malignancies leads to mitochondrial anomalies, promoting cancer cell survival, migration and invasion. Although environmental factors are implicated in cancer, genetic components play a substantial role, with heritability estimates from family studies ranging between 27% and 64%.^10^, ^11^

In the context of immunotherapy, the tumour mutational burden (TMB)—the quantification of somatic mutations—has emerged as a crucial prognostic and predictive biomarker for anti‐programmed cell death (PD) 1/anti‐PD‐ligand 1 treatment and other immunotherapeutic drugs.^12^ Tumour‐infiltrating lymphocytes and TMB are recognised as primary molecular predictors for immunotherapeutic efficacy and survival advantage in various cancers.^13^ Studies have shown that first‐degree relatives of women with CESC have up to a threefold increased risk for the disease [18]. Moreover, mtDNA mutations are closely associated with the TME, HPV infection and HPV types, underscoring their significance.^14^, ^15^ However, the role of MTRGs and TME in determining CESC prognosis is still not fully understood.

The lack of effective treatments specifically targeting stage IV mtDNA mutations in recurrent or metastatic cases underscores the potential of immunotherapy as a promising avenue for patients with intractable CESC.^16^, ^17^ Identifying and targeting high‐risk germline mutations could significantly improve the survival rates of cancer patients [23]. Accurately identifying individuals with solid tumours who are likely to respond to immunotherapy remains a critical challenge, paving the way for advancements in personalised cancer treatment.

Recent advancements in the understanding of CESC have led to the development of various prognostic models, primarily focusing on genetic alterations, expression patterns and clinical features. However, despite these advancements, the prognostic accuracy of these models remains limited, particularly in predicting long‐term outcomes and treatment responses. This limitation underscores the urgent need for innovative biomarkers and therapeutic targets that can enhance the precision of prognostic models and guide the development of effective immunotherapeutic strategies. Mitochondria play a crucial role in the TME during cancer progression, influencing metabolic reprogramming, immune evasion and resistance mechanisms. Given this significance, our study aims to investigate the potential of MTRGs and TME factors in constructing a predictive model for CESC. By identifying and characterizing novel biomarkers and examining their interplay with immune infiltration in the TME, we aim to develop an innovative prognostic model that can improve the prediction of CESC outcomes. This approach not only holds promise for advancing our understanding of the molecular underpinnings of CESC but also for guiding personalised immunotherapeutic interventions.

## 2 MATERIALS AND METHODS

### 2.1 Accessing and Preprocessing of CESC Datasets

The expression datasets of Cervical Squamous Cell Carcinoma (CESC) samples were collected from The Cancer Genome Atlas (TCGA) and Genotype‐Tissue Expression (GTEx) databases (https://xenabrowser.net/datapages/). Simultaneously, clinical follow‐up data for these CESC samples was obtained. Samples lacking clinical data and those with a follow‐up time of less than 30 days were excluded. Additionally, the 147 mitochondria‐related genes (MTRGs) were retrieved from the MITOMAP database for the human mitochondrial genome (http://www.MITOMAP.org), and all MTRGs were integrated into a nuclear mitochondria‐related gene set. The probe names and annotations of all microarray datasets were standardized. For probes associated with the same gene name, the median expression level of the gene was calculated as the representative gene expression value using the R software package Bioconductor. Multiple probes matching the median expression of a gene were considered. The data underwent normalisation before any further analysis.

### 2.2 Differentially Expressed Genes (DEGs) Related to CESC

RNA‐sequencing data comparing CESC samples and normal individuals were extracted from the database to identify differentially expressed genes (DEGs) associated with mitochondria [|log2‐fold change (FC)| >1, *q*‐value < 0.01].

To establish a prognostic mitochondrial (MT) score model and conduct survival analysis, we employed R Studio. Initially, a univariate Cox proportional hazards regression analysis was performed on the TCGA cervical cancer dataset to assess the correlation between changes in mitochondria‐related genes (MTRGs) and the survival of CESC patients.

The “glmnet” package in R Studio was then utilised for least absolute shrinkage and selection operator (LASSO) Cox regression with 10‐fold cross‐validation, aiming to eliminate unfavourable genes.^18^ Through univariate Cox analysis, 94 mitochondria‐related DEGs were identified in the TCGA dataset, with 14 MTRGs closely associated with prognosis. Subsequently, multivariate Cox regression analysis was applied to construct an MT score model and identify prognostic MTRGs using bootstrap samples.

The MT score was calculated as the sum of each MTRG expression value multiplied by its coefficient in the multivariate Cox regression model. Using the median MT score as a cut‐off threshold, CESC patients were categorised into two groups: those with MT high and low subtypes.

The “survival” package in R Studio was employed to construct Kaplan–Meier (KM) curves. Additionally, the “timeROC” package in R Studio calculated the area under the ROC curve (AUC) to evaluate the prognostic capacity of the identified MTRG signature. Univariate and multivariate Cox regression analyses were conducted to determine the prognostic values of the signature. The “rms” package in R was utilised to create nomograms and calibration graphs.

### 2.3 Evaluation of the Immune Cell Infiltration and TME analysis of CESC samples

CIBERSORT is a method that applies the linear support vector regression theory to deconvolute the expression matrix of immune cell subtypes. Using RNA sequencing data, it can determine the type percentage in large tumour samples with admixed cell types.^19^ We exploited the CIBERSORT algorithm and its LM22 gene signature to calculate the proportions of immune cells and survey the infiltrating immune cells in TME between MT high and low cohorts, which can sensitively and specifically discriminate 22 human immune cell phenotypes, including B cells, T cells, natural killer (NK) cells, monocytes, macrophages, dendritic cells (DCs), mast cells, neutrophils, eosinophils, etc.^20^ In addition, to further elucidate the infiltrating lymphocytes, the stromal score, the immunological score and the ESTIMATE score were computed.^21^ The standardisation data of gene expression were loaded into the CIBERSORT website. (https://ciberfort.stanford.edu/). Next, we set the cut‐off to 0.05 and then eliminated those samples that did not meet the threshold. The scores of 22 immune cells were determined using LM22 signature and 1000 permutation.

### 2.4 Construction of a Risk Score Model Based on TME score and Survival Analysis

The abundance of 22 immune cells in TCGA cohorts was calculated by CIBERSORT, and immune cells significantly related to prognosis were screened out by KM prognostic analysis. At the same time, multivariate Cox regression analysis was further used to identify prognostic TME and to construct a TME score model based on bootstrap samples. According to the TME score, CESC patients were divided into two subgroups; those with TME high and low subtypes, respectively.

### 2.5 Enrichment Analyses of Gene Set and KEGG Pathway

Using the “clusterProfifiler” package in R Studio, the gene set enrichment analysis (GSEA) was evaluated to ascertain whether there were significant differences in the list of genes expressed and common pathways involved during CESC progression and metastasis between MT and TME distinct subtypes.^22^ We primarily centred on Kyoto Encyclopedia of Genes and Genomes (KEGG) (KEGG, https://www.kegg.jp/) pathway enrichment analysis and Hallmark gene set enrichment analysis (http://www.gseamsigdb.org/gsea/), which were both plotted using the “ggplot2” package in R Studio. The cut‐off was set at a *p*‐value of less than 0.05.

Development of a Classifier on the Premise of MT in Conjunction with TME Score via Machine Learning (ML) Tool.

Deep learning is a subfield of artificial intelligence that employs artificial neural networks, a machine learning approach, to discover patterns and forecast outcomes from massive data sets. Numerous research teams have investigated the application of machine learning (ML) approaches due to the significance of categorising cancer patients into high‐ and low‐risk categories, which have been utilised as an aim to model the progression and treatment of cancerous conditions.^23^, ^24^, ^25^, ^26^ In this work, machine learning was used to develop the MT score and TME score systems. In the pathway analysis of genes related to two score systems, we discovered a definite association between two score systems. The MT‐TME classifier was constructed based on the MT score and TME score systems, and the OS curve analysis of the four types was also performed in the classifier.

### 2.6 Construction of Co‐Expression Modules of CESC by WGCNA

The co‐expression modules were identified using the weighted gene co‐expression network analysis (WGCNA) method in the R software package (http://www.r‐project.org/). The intensity of the interactions was examined using the heatmap tools package. In this study, core modules and central genes related to CESC were identified via WGCNA in MT‐TME classifier. Enrichment analyses of the identified modules in MT‐TME classifier were performed using a Metascape dataset.

### 2.7 Effect Analysis of Immunotherapy in MT‐TME classifier

TMB differences were analysed in each subtype of the MT‐TME classifier, and OS curves were generated for each subtype combined with TMB high and low subtypes. Additionally, we evaluated the disparate patterns of tumour cell mutations in patients with the MT and TME subgroups, as well as the disparate immunotherapy response rates of each subgroup in MT‐TME classifier and distinct MT score and TME score systems.

### 2.8 Proteomic and Functional Analysis in MT‐TME classifier

The expression and prognosis of proteins were generated utilising the CPTAC proteomics database (https://cptac‐data‐portal.georgetown.edu/cptacPublic/). Moreover, the proteomics and phospho‐proteomics data among patients were also downloaded. These data could effectively verify the association between proteins and distinct subtypes in MT‐TME classifier and identify candidate proteins that might be employed as biomarkers for tumours.^27^

### 2.9 Statistical analysis

We performed all analyses and visualisation in R software (version 4.2.1), unless otherwise stated.

## 3 RESULTS

### 3.1 Acquisition of DEGs and construction of a risk model based on of MT score

**FIGURE 1:**
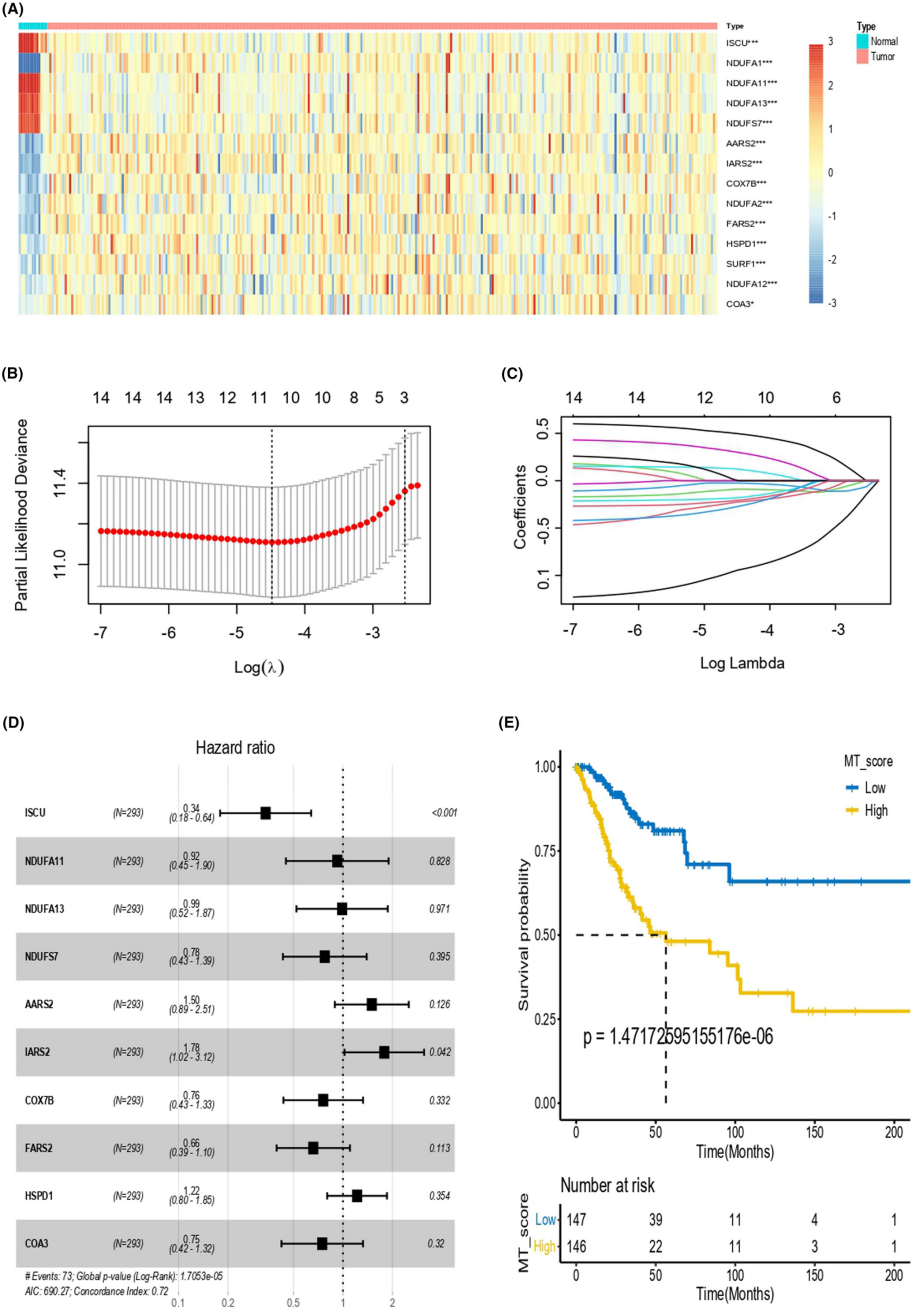
DEGs among MTRGs and the construction of MT score model. (A) Heatmap exhibited mitochondria related genes strongly associated with CESC prognosis. (B, C) The Lasso model was established to eliminate undesirable genes. (D) The MT score system was constructed based on bootstrap samples by multicox regression coefficients. (E) The overall survival curve showed survival possibility between high and low MT score subgroups in CESC.

In this study, we obtained 147 MTRG data, and a total of 94 DEGs related to mitochondria were identified in TCGA database, among which 14 genes were correlated with prognosis were selected by univariate Cox regression analysis from the TCGA CESC dataset, including ISCU, NDUFA11, NDUFA13, NDUFS7, NDUFA1, AARS2, IARS2, COX7B, NDUFA2, FARS2, HSPD1, SURF1, NDUFA12 and COA3 (Figure 1A). Additionally, the least absolute shrinkage and selection operator (Lasso) model was developed to get rid of undesirable genes (Figure 1B,C).

Next, multivariate Cox (multiCox) regression analysis was performed to develop the MT score model based on bootstrap samples (Figure 1D). The OS curves were depicted between the MT low score (*n* = 147) and MT high score (*n* = 146) cohorts in CESC, which exhibited that the former was strongly connected to a favourable prognosis while the latter was greatly related to a negative prognosis (Figure 1E).

### 3.2 Establishment of the TME score model

**FIGURE 2:**
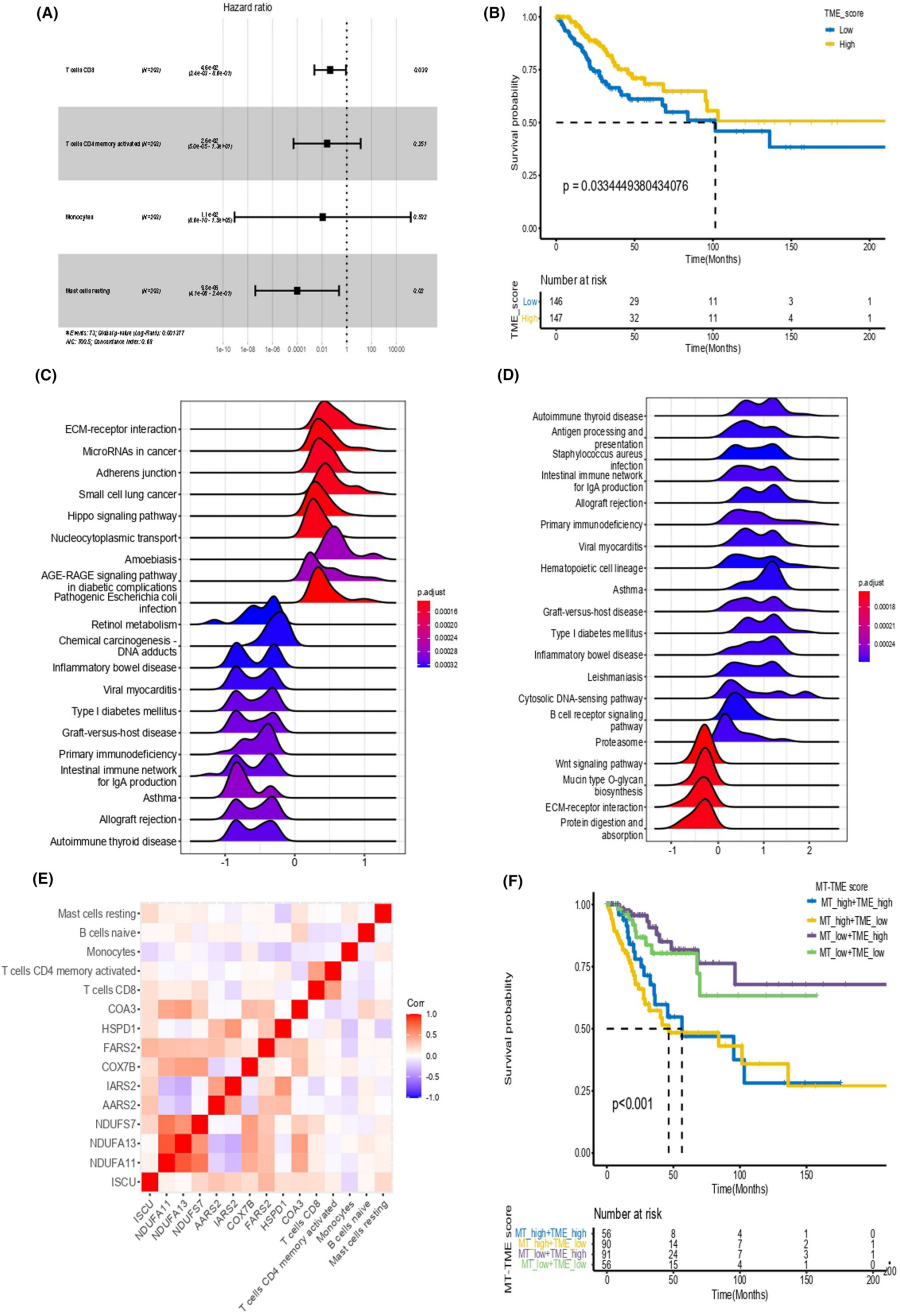
Establishment of TME score model and MT‐TME classifier. (A) Forest plots showed the role of immune cells in relation to prognosis. (B) The overall survival curves of the TME low and high score cohorts in the TME prognostic model. (C, D) GSEA enrichment analyses were performed in MT score and TME score groups, respectively. (E) Analysis of correlation revealed the link between MT and TME scores. (F) Overall survival trend of each subtype in the MT‐TME classifier in CESC.

In TCGA datasets, the abundance of 22 immune cells was computed using CIBERSORT, and the immune cells substantially related with the prognosis were identified using Kaplan–Meier (KM) analysis, including B cells native, CD8 T cells, activated CD4 T cells, regulatory T cells, macrophages M0, activated mast cells, resting mast cells, monocytes and neutrophils (Figure S1). The forest map highlighted the crucial role that associated immune cells played in CESC, particularly CD8 T cells and mast cells resting (Figure 2A). In addition, we created a TME score model that was linked with prognosis by computing model coefficients using multicox regression on bootstrap data. Overall survival curves correlated with clinical prognosis were displayed between TME high and low subtypes, demonstrating that the former subtype had a considerably better prognosis than the later subtype (Figure 2B). Then, GSEA enrichment analyses were performed respectively on the MT and TME score cohorts, where the former was particularly enriched in ECM‐receptor interaction, MicroRNAs in cancer, adherens junction and Hippo signalling pathway, and the latter was considerably enriched in Wnt signalling pathway, mucin‐type O‐glycan biosynthesis pathway, ECM‐receptor interaction and protein digestion and absorption (Figure 2C, D).

### 3.3 Development of the MT‐TME classifier

The MT score and TME score systems were developed through machine learning. In the pathway analysis of core genes between the two score systems, MT score was strongly correlated with TME score (Figure 2C–E). Correlation analysis revealed that monocytes were negatively associated with HSPD1 and FARS2, while ISCU was favourably correlated with CD8 T cells and negatively correlated with monocytes (Figure 2E). In addition, MT‐TME classifier was developed based on the MT and TME score model. The OS curves of four subtypes in classifiers were presented, and the combination of MT low‐TME high subtype had the best prognosis, while the MT high‐TME low subtype had the worst prognosis (Figure 2F). Consequently, the MT‐TME classifier may be valuable for predicting OS and needs additional investigation.

### 3.4 Functional analyses of MT‐TME classifier

**FIGURE 3:**
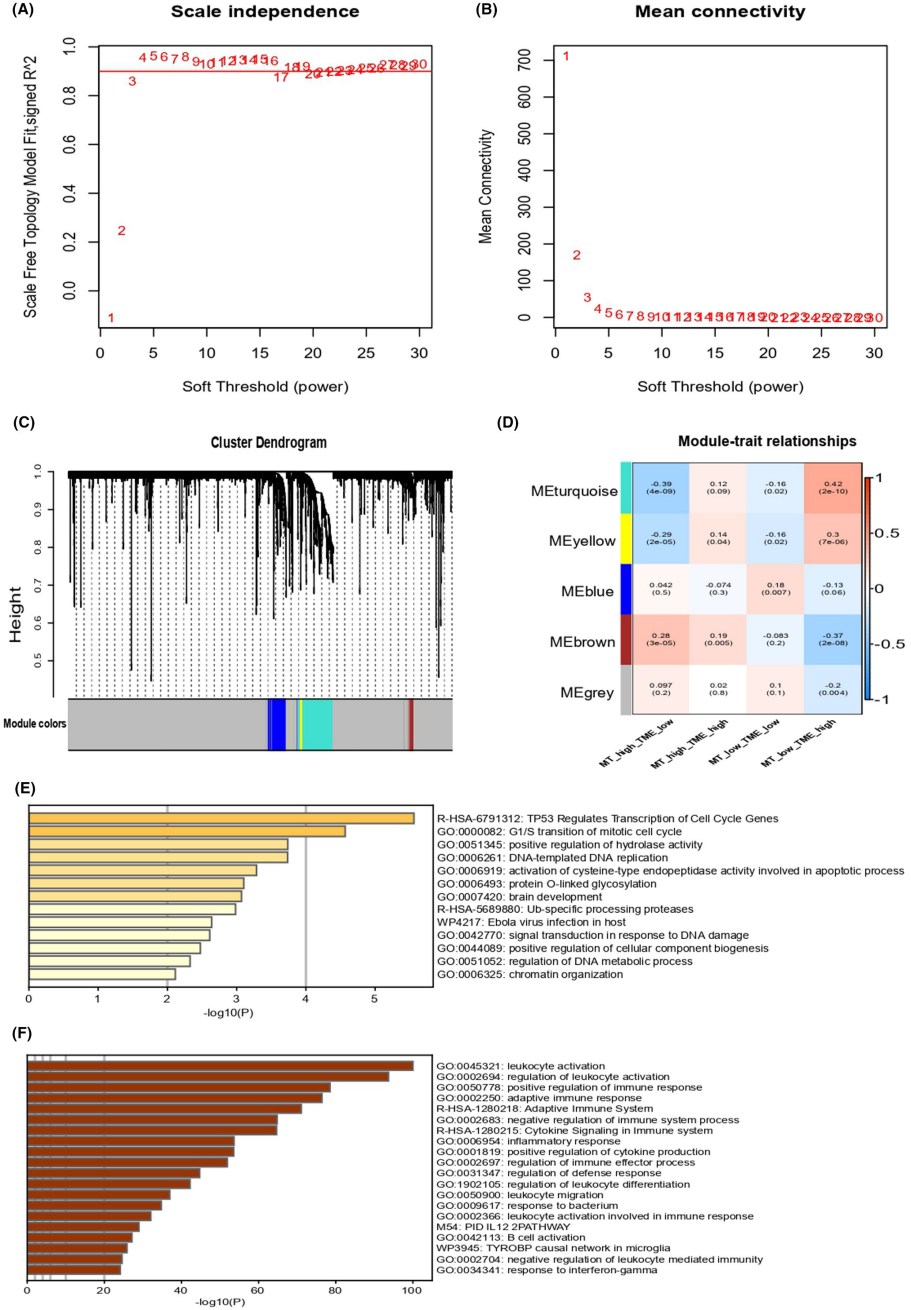
Development of Co‐expression modules of CESC by WGCNA. (A–D)The graph depicted the connection between each MT‐TME classifier module. (E, F) The diagram illustrated the enrichment analysis of MT‐TME classifier modules.

Co‐expression networks were constructed using WGCNA (Figure 3). There were a total of five modules found in MT‐TME classifier, where brown module had a positive relationship with MT high‐TME low subgroup and a negative correlation with MT low‐TME high subgroup, whereas turquoise and yellow module had a negative correlation with MT high‐TME low subgroup and a positive relationship with MT low‐TME high subgroup (Figure 3A–D). Using Metascape, we analysed the enrichment of genes in brown, turquoise and yellow modules. The corresponding module‐related genes were all enriched in immune and evolution‐related pathways between MT high‐TME low and MT low‐TME high subgroup, indicating that distinct immunological abnormalities were hallmarks of the pathological process with CESC, and mitochondria could play a crucial role in this process (Figure 3E, F). Due to poor predictive capabilities, MT high‐TME high subgroup and MT low‐TME low subgroup were amalgamated to form the mixed subgroup. Fast GSEA was then performed to evaluate enrichment in MT low‐TME high subtype, MT high‐TME low subtype and the mixed subtype (Figure S2).

### 3.5 Comparative analysis of gene expression, mutation patterns and immune checkpoint profiles in MT‐TME subgroups of CESC

**FIGURE 4:**
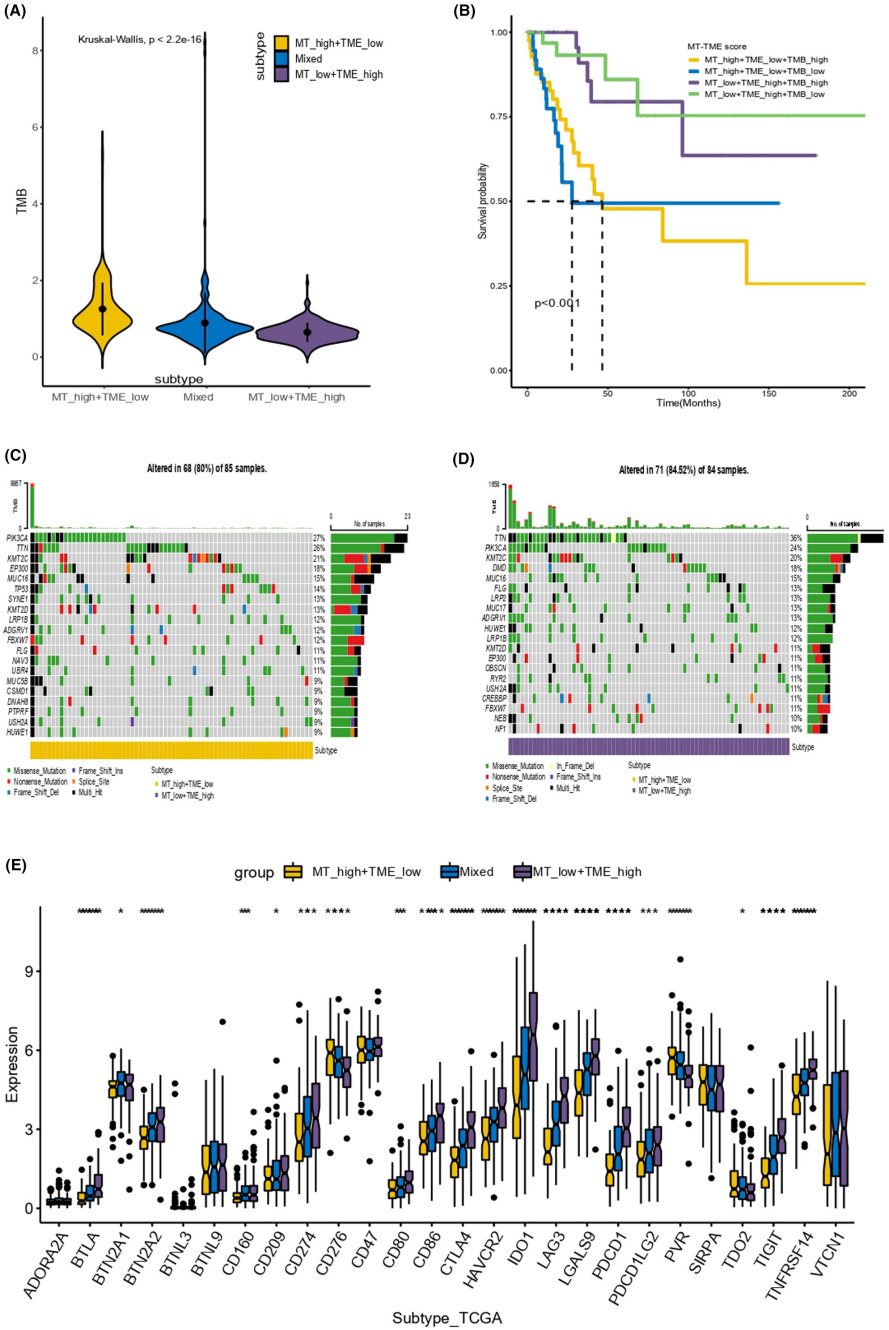
Effect of immunotherapy in the MT‐TME classifier. (A) TMB difference analyses of three groups in MT‐TME classifier. (B) The overall survival curve showed survival probability of MT‐TME classifier combined with TMB high and low classification in CESC. (C, D) The diagram depicted differential pattern analyses of tumour cell mutations in the MT and TME subgroups. (E) The figure exhibited differential analysis of common immune checkpoints.

**FIGURE 5:**
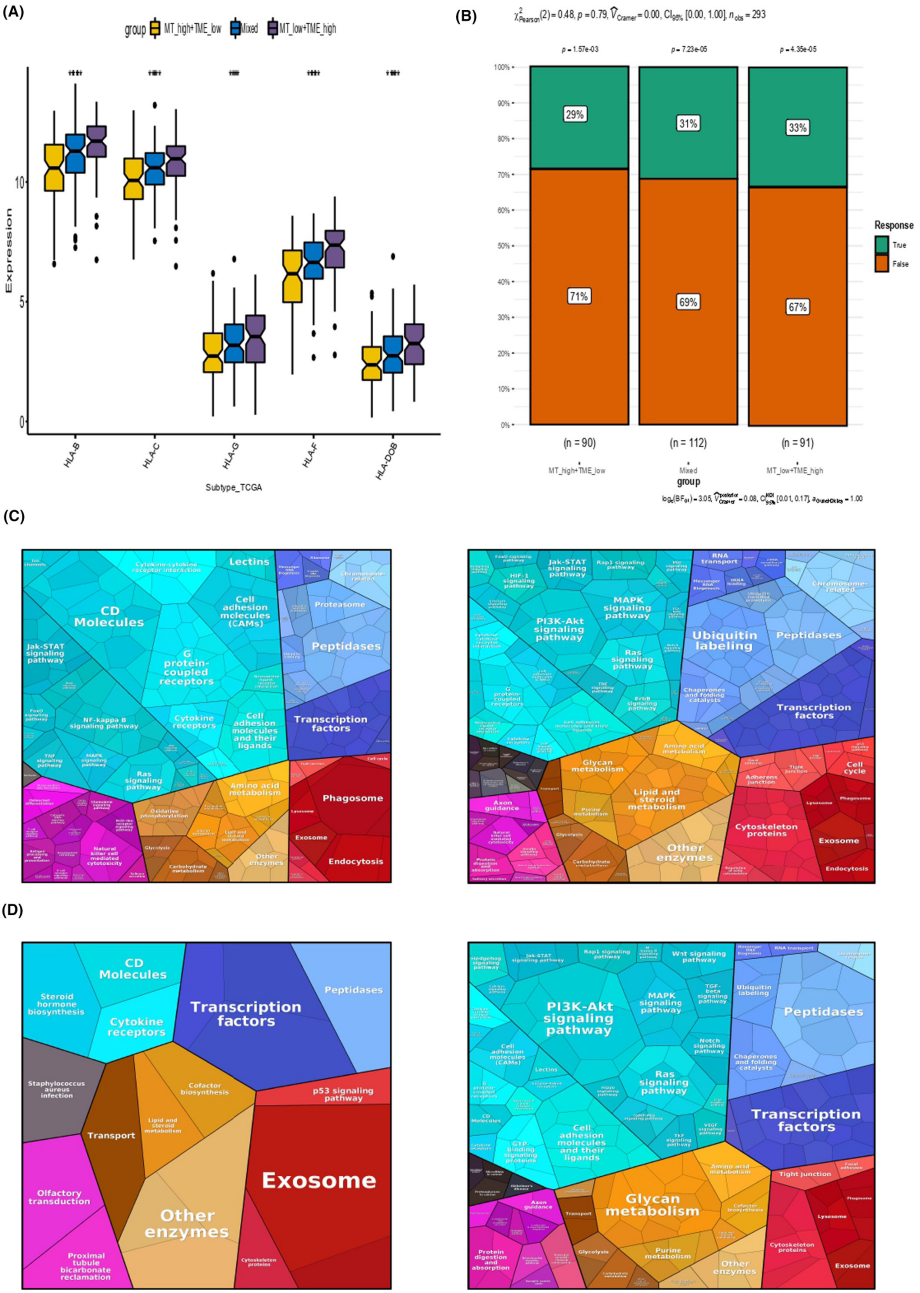
Difference analysis of immunotherapy response rate and key protein function signalling pathway among subgroups. (A, B) Analysis of the differences in immunotherapy response of each subgroup in MT‐TME classifier and the differences between the two score systems. (C) Differences analyses in key protein functional signalling pathways between MT High‐TME low (left) and MT Low‐TME high (right). (D) Differences analyses in key protein functional signalling pathways between high and low immune response groups.

The variation analyses in gene expression were performed among MT high‐TME low subtype, MT low‐TME high subtype and the mixed subtype (MT high‐TME high and MT low‐TME low) (Figure 4A). The OS curves of MT‐TME classifier combined with TMB subgroups in CESC were displayed (Figure 4B). Besides, we analysed the distinct tumour cell mutation patterns in MT‐TME subgroups (Figure 4C, D). In MT high‐TME low subgroup, tumour mutations were identified in 68 of 85 samples (80%), including PIK3CA (27%), TTN (26%) and KMT2C (21%). In MT low‐TME high subgroup, gene alterations were detected in 71 of 84 samples (84.52%), including TTN (36%), PIK3CA (24%) and KMT2C (20%). Moreover, the results also exhibited the difference analysis at common immune checkpoints across three subtypes in MT‐TME classifier, where ADORA2A, BTN2A2, CD160, CD274, CD276, CD86, CTLA4, HAVCR2, IDO1, LAG3, LGALS9, PDCD1, PDCD1LG2, PVR, TIGIT and TNFRSF14 were statistically significant. Furthermore, the differential study of key immune checkpoints was performed among three subgroups (Figure 4E). Additionally, Figure 5A,B demonstrated the difference analyses of immunotherapy response rate in each subgroup of MT‐TME classifier and the difference between MT and TME score systems (Figure 5A, B).

### 3.6 Proteomic function analysis of MT and TME score system

Proteomic functional analysis elucidated significant distinctions in the primary protein signalling pathways between the subgroups characterised by MT activity and low TME involvement (left side) and those with low MT activity but high TME involvement (right side). This divergence was also pronounced when comparing subgroups with high versus low immune responses, as illustrated in Figure 5D.

## 4 DISCUSSION

Cervical squamous cell carcinoma (CESC) stands as a prevalent gynaecological concern globally. Recognised risk factors, such as parity, high‐risk HPV infection, smoking, alcohol use, multiple sexual partners and oral contraceptives, significantly influence CESC development and progression.^28^ Late‐stage detection leads to poor prognosis and high mortality despite advancements in early detection techniques and therapeutic improvements, highlighting the need for accurate prognostic models in CESC management.^6^

Mitochondria are increasingly recognized for their pivotal role in cancer cell metabolism, with mitochondrial dysfunction identified as a key hallmark in various diseases and influenced by numerous factors, including disease states and adverse environmental conditions.^29^, ^30^, ^31^ This dysfunction, often resulting from MtDNA mutations, deletions and impairments in DNA replication, significantly contributes to cancer pathogenesis by promoting resistance to apoptosis, uncontrolled cell proliferation and treatment resistance.^32^, ^33^, ^34^, ^35^ Dysregulation of mitophagy, involving proteins such as Parkin, PINK1, BNIP3, BNIP3L/NIX and p62/SQSTM1, further exacerbates tumour growth and metastasis by fueling cancer cell energy demands and enhancing cell survival through anti‐apoptotic pathways.^36^, ^37^ This process is crucial for maintaining the delicate balance between aerobic glycolysis and oxidative phosphorylation, which is vital for cancer cell viability.^33^ Recent research has also implicated increased mitochondrial fission as a tumour‐promoting factor, emphasising the critical role of mitochondrial dynamics in cancer progression.^38^, ^39^

Several MTRGs are implicated in tumour development, growth, metastasis and recurrence. Given the pivotal role of mitochondria in cancer development, recent studies suggest that MTRGs could serve as potential biomarkers for CESC diagnosis and prognosis, as well as targets for immunological or molecular therapies related to mitochondrial dysfunction.^36^, ^40^ However, the applicability of MTRGs in CESC remains elusive, underscoring the need to explore their relationship for developing effective immunotherapy approaches in cancer treatment.

In this study, we identified 147 MTRGs, pinpointing 94 mitochondria‐related lineage‐associated DEGs. Subsequent multivariate Cox regression analysis identified 14 mitochondria‐related core genes, including NDUFA1, AARS2, IARS2, COX7B, NDUFA2, FARS2, HSPD1, SURF1, NDUFA12, COA3, ISCU, NDUFA11, NDUFA13 and NDUFS7, as key predictors in a prognostic risk model for predicting overall survival (OS) in cervical cancer patients. Notably, high expression of certain genes represented high‐risk and worse prognosis, while high expression of others was associated with lower risk in CESC.

For instance, ISCU, a target gene of miR‐210, demonstrated an inverse link with prognosis and was down‐regulated by miR‐210 in vivo, leading to tumour development. The study revealed potential therapeutic targets based on somatic mutations of ISCU in CESC tissues.^41^, ^42^ The mRNA expression of NDUFS7, linked to survival state and poor prognosis, was notably lower in cancer patients than in normal tissue, suggesting its potential utility in identifying high‐risk cancer patients. Additionally, COX7B, IARS2, COA3 and NDUFA1 were associated with platinum resistance, cancer cell proliferation and invasion, highlighting their significance as potential therapeutic targets and prognostic biomarkers.^43^, ^44^, ^45^, ^46^, ^47^, ^48^

Persistent high‐risk HPV infection and inadequate immune response are pivotal pathogenic factors in cervical cancer.^49^, ^50^ The study investigated 22 immune cell types, revealing significant roles of 10 immune cell types in cervical cancer prognosis. Further analyses, including WGCNA, highlighted substantial immune changes in specific subgroups, emphasizing the intricate relationship between the immunological environment and mitochondria.

This study, while providing valuable insights into the role of MTRGs in cervical cancer, is not without limitations. Firstly, the retrospective nature of our analysis, relying on previously collected data, may introduce biases that could affect the generalizability of our findings. Secondly, our study is constrained by the lack of experimental validation for the identified MTRGs, which means the functional roles and mechanisms of these genes in the TME remain hypothetical. Additionally, our analysis is based on a single dataset, which may limit the robustness and applicability of our results across diverse populations and clinical settings. Furthermore, the complexity of the TME and the myriad interactions between tumour cells, immune cells and other stromal components are not fully captured in our study, potentially oversimplifying the intricate dynamics at play. Lastly, while our study suggests potential therapeutic targets, the clinical utility and efficacy of targeting these MTRGs in cervical cancer treatment need to be rigorously evaluated in prospective clinical trials.

In conclusion, our research highlights the profound impact of MTRGs on the TME and immune response in cervical cancer. By delineating distinct proteomic signatures across different subgroups, we have uncovered the pivotal role of MTRGs in modulating tumour behaviour and response to therapy. These insights not only enhance our understanding of cervical cancer pathogenesis but also open new avenues for personalised treatment approaches, emphasising the potential of MTRGs as valuable prognostic tools and therapeutic targets.

## AUTHOR CONTRIBUTIONS

**Lingxiao Ying:** Conceptualization (equal); writing – original draft (equal); writing – review and editing (equal). **Lin Kong:** Data curation (equal). **Xiaoxiao Qiu:** Data curation (equal). **Aihua Cheng:** Data curation (equal). **Qijun Wang:** Data curation (equal). **Limeng Xiu:** Data curation (equal). **Jinmei Shi:** Data curation (equal). **Yanfei Tao:** Data curation (equal). **Zhihong Chai:** Resources (equal).

## FUNDING INFORMATION

2022 Zhejiang Provincial Health Department Project “Mechanistic Study on HPV16 Cell‐Penetrating Peptide Enhancing the Immunogenicity of HPV16 L2 Peptide” (2022PY104). 2022 Ministry of Education Industry‐University Cooperation Collaborative Education Project “Study on the Inhibition of Cervical Cancer Cell Proliferation and Glucose Metabolism by Recombinant Virus‐Like Particle Protein L2 (RVL2) through the IT GB7/C/EBPβ Signaling Pathway” (220604408300411).

## CONFLICT OF INTEREST STATEMENT

None declared.

## Supporting information


Figure S1.

Figure S2.

## Data Availability

The original contributions presented in the study are included in the article/supplementary material, further inquiries can be directed to the corresponding author.

## References

[1] Bray F , Ferlay J , Soerjomataram I , Siegel RL , Torre LA , Jemal A . Global cancer statistics 2018: GLOBOCAN estimates of incidence and mortality worldwide for 36 cancers in 185 countries. CA Cancer J Clin. 2018;68(6):394‐424. 30207593 10.3322/caac.21492

[2] Arbyn M , Weiderpass E , Bruni L , et al. Estimates of incidence and mortality of cervical cancer in 2018: a worldwide analysis. Lancet Glob Health. 2020;8(2):e191‐e203. 31812369 10.1016/S2214-109X(19)30482-6 PMC7025157

[3] Liao JB , Fisher CE , Madeleine MM . Gynecologic cancers and solid organ transplantation. Am J Transplant. 2019;19(5):1266‐1277. 30725527 10.1111/ajt.15292

[4] Crosbie EJ , Einstein MH , Franceschi S , Kitchener HC . Human papillomavirus and cervical cancer. Lancet. 2013;382(9895):889‐899. 23618600 10.1016/S0140-6736(13)60022-7

[5] Canfell K , Kim JJ , Brisson M , et al. Mortality impact of achieving WHO cervical cancer elimination targets: a comparative modelling analysis in 78 low‐income and lower‐middle‐income countries. Lancet. 2020;395(10224):591‐603. 32007142 10.1016/S0140-6736(20)30157-4 PMC7043006

[6] Moore DH . Cervical cancer. Obstet Gynecol. 2006;107(5):1152‐1161. 16648423 10.1097/01.AOG.0000215986.48590.79

[7] Xiao Z , Dai Z , Locasale JW . Metabolic landscape of the tumor microenvironment at single cell resolution. Nat Commun. 2019;10(1):3763. 31434891 10.1038/s41467-019-11738-0 PMC6704063

[8] Kaur P , Nagar S , Bhagwat M , et al. Activated heme synthesis regulates glycolysis and oxidative metabolism in breast and ovarian cancer cells. PloS One. 2021;16(11):e0260400. 34807950 10.1371/journal.pone.0260400 PMC8608300

[9] Chen B , Zhang G , Li X , et al. Comparison of BRCA versus non‐BRCA germline mutations and associated somatic mutation profiles in patients with unselected breast cancer. Aging. 2020;12(4):3140‐3155. 32091409 10.18632/aging.102783 PMC7066887

[10] Hemminki K , Chen B . Familial risks for cervical tumors in full and half siblings: etiologic apportioning. Cancer Epidemiol Biomarkers Prev. 2006;15(7):1413‐1414. 16835346 10.1158/1055-9965.EPI-05-0933

[11] Bowden SJ , Bodinier B , Kalliala I , et al. Genetic variation in cervical preinvasive and invasive disease: a genome‐wide association study. Lancet Oncol. 2021;22(4):548‐557. 33794208 10.1016/S1470-2045(21)00028-0 PMC8008734

[12] McNamara MG , Jacobs T , Lamarca A , Hubner RA , Valle JW , Amir E . Impact of high tumor mutational burden in solid tumors and challenges for biomarker application. Cancer Treat Rev. 2020;89:102084. 32738738 10.1016/j.ctrv.2020.102084

[13] Yan J , Wu X , Yu J , Zhu Y , Cang S . Prognostic role of tumor mutation burden combined with immune infiltrates in skin cutaneous melanoma based on multi‐omics analysis. Front Oncol. 2020;10:570654. 33240814 10.3389/fonc.2020.570654 PMC7683772

[14] Zhu Z , Wang X . Significance of mitochondria DNA mutations in diseases. Adv Exp Med Biol. 2017;1038:219‐230. 29178079 10.1007/978-981-10-6674-0_15

[15] Gammage PA , Frezza C . Mitochondrial DNA: the overlooked oncogenome? BMC Biol. 2019;17(1):53. 31286943 10.1186/s12915-019-0668-y PMC6615100

[16] Ferlay J , Colombet M , Soerjomataram I , et al. Estimating the global cancer incidence and mortality in 2018: GLOBOCAN sources and methods. Int J Cancer. 2019;144(8):1941‐1953. 30350310 10.1002/ijc.31937

[17] Zhu S , Zhang T , Zheng L , et al. Combination strategies to maximize the benefits of cancer immunotherapy. J Hematol Oncol. 2021;14(1):156. 34579759 10.1186/s13045-021-01164-5 PMC8475356

[18] Wang H , Lengerich BJ , Aragam B , Xing EP . Precision Lasso: accounting for correlations and linear dependencies in high‐dimensional genomic data. Bioinformatics. 2019;35(7):1181‐1187. 30184048 10.1093/bioinformatics/bty750 PMC6449749

[19] Gentles AJ , Newman AM , Liu CL , et al. The prognostic landscape of genes and infiltrating immune cells across human cancers. Nat Med. 2015;21(8):938‐945. 26193342 10.1038/nm.3909 PMC4852857

[20] Li T , Fu J , Zeng Z , et al. TIMER2.0 for analysis of tumor‐infiltrating immune cells. Nucleic Acids Res. 2020;48(W1):W509‐W514. 32442275 10.1093/nar/gkaa407 PMC7319575

[21] Yoshihara K , Shahmoradgoli M , Martínez E , et al. Inferring tumour purity and stromal and immune cell admixture from expression data. Nat Commun. 2013;4:2612. 24113773 10.1038/ncomms3612 PMC3826632

[22] Yu G , Wang LG , Han Y , He QY . clusterProfiler: an R package for comparing biological themes among gene clusters. OMICS. 2012;16(5):284‐287. 22455463 10.1089/omi.2011.0118 PMC3339379

[23] Liu J , Chen P , Zhou J , Li H , Pan Z . Prognostic impact of lactylation‐associated gene modifications in clear cell renal cell carcinoma: insights into molecular landscape and therapeutic opportunities. Environ Toxicol. 2024;39(3):1360‐1373. 37972232 10.1002/tox.24040

[24] Tran KA , Kondrashova O , Bradley A , Williams ED , Pearson JV , Waddell N . Deep learning in cancer diagnosis, prognosis and treatment selection. Genome Med. 2021;13(1):152. 34579788 10.1186/s13073-021-00968-x PMC8477474

[25] Kourou K , Exarchos TP , Exarchos KP , Karamouzis MV , Fotiadis DI . Machine learning applications in cancer prognosis and prediction. Comput Struct Biotechnol J. 2015;13:8‐17. 25750696 10.1016/j.csbj.2014.11.005 PMC4348437

[26] Li H , Zhou L , Zhou W , et al. Decoding the mitochondrial connection: development and validation of biomarkers for classifying and treating systemic lupus erythematosus through bioinformatics and machine learning. BMC Rheumatol. 2023;7(1):44. 38044432 10.1186/s41927-023-00369-0 PMC10694981

[27] Tabb DL , Wang X , Carr SA , et al. Reproducibility of differential proteomic technologies in CPTAC fractionated xenografts. J Proteome Res. 2016;15(3):691‐706. 26653538 10.1021/acs.jproteome.5b00859 PMC4779376

[28] Burd EM . Human papillomavirus and cervical cancer. Clin Microbiol Rev. 2003;16(1):1‐17. 12525422 10.1128/CMR.16.1.1-17.2003 PMC145302

[29] Lee AS . Glucose‐regulated proteins in cancer: molecular mechanisms and therapeutic potential. Nat Rev Cancer. 2014;14(4):263‐276. 24658275 10.1038/nrc3701 PMC4158750

[30] Wu Y , Zhang S , Gong X , et al. The epigenetic regulators and metabolic changes in ferroptosis‐associated cancer progression. Mol Cancer. 2020;19(1):39. 32103754 10.1186/s12943-020-01157-x PMC7045519

[31] Zong WX , Rabinowitz JD , White E . Mitochondria and cancer. Mol Cell. 2016;61(5):667‐676. 26942671 10.1016/j.molcel.2016.02.011 PMC4779192

[32] Wallace DC . Mitochondria and cancer. Nat Rev Cancer. 2012;12(10):685‐698. 23001348 10.1038/nrc3365 PMC4371788

[33] Burke PJ . Mitochondria, bioenergetics and apoptosis in cancer. Trends Cancer. 2017;3(12):857‐870. 29198441 10.1016/j.trecan.2017.10.006 PMC5957506

[34] Seervi M , Sumi S , Chandrasekharan A , Sharma AK , SanthoshKumar TR . Molecular profiling of anastatic cancer cells: potential role of the nuclear export pathway. Cell Oncol (Dordr). 2019;42(5):645‐661. 31147963 10.1007/s13402-019-00451-1 PMC12994364

[35] Li H , Zhou L , Zhou W , et al. Mitochondrial aberrations in systemic lupus erythematosus pathogenesis: insights and therapeutic implications. Rheumatol Autoimmunity. 2024.

[36] Panigrahi DP , Praharaj PP , Bhol CS , et al. The emerging, multifaceted role of mitophagy in cancer and cancer therapeutics. Semin Cancer Biol. 2020;66:45‐58. 31351198 10.1016/j.semcancer.2019.07.015

[37] Wang SF , Chen S , Tseng LM , Lee HC . Role of the mitochondrial stress response in human cancer progression. Exp Biol Med (Maywood). 2020;245(10):861‐878. 32326760 10.1177/1535370220920558 PMC7268930

[38] Chourasia AH , Tracy K , Frankenberger C , et al. Mitophagy defects arising from BNip3 loss promote mammary tumor progression to metastasis. EMBO Rep. 2015;16(9):1145‐1163. 26232272 10.15252/embr.201540759 PMC4576983

[39] Capparelli C , Guido C , Whitaker‐Menezes D , et al. Autophagy and senescence in cancer‐associated fibroblasts metabolically supports tumor growth and metastasis via glycolysis and ketone production. Cell Cycle. 2012;11(12):2285‐2302. 22684298 10.4161/cc.20718 PMC3383590

[40] Srinivasan S , Guha M , Kashina A , Avadhani NG . Mitochondrial dysfunction and mitochondrial dynamics‐the cancer connection. Biochimica Biophys Acta Bioenerg. 2017;1858(8):602‐614. 10.1016/j.bbabio.2017.01.004 PMC5487289 28104365

[41] Gee HE , Ivan C , Calin GA , Ivan M . HypoxamiRs and cancer: from biology to targeted therapy. Antioxid Redox Signal. 2014;21(8):1220‐1238. 24111776 10.1089/ars.2013.5639 PMC4142802

[42] Shi J , Wu P , Sheng L , Sun W , Zhang H . Ferroptosis‐related gene signature predicts the prognosis of papillary thyroid carcinoma. Cancer Cell Int. 2021;21(1):669. 34906147 10.1186/s12935-021-02389-7 PMC8670268

[43] Tanaka N , Katayama S , Reddy A , et al. Single‐cell RNA‐seq analysis reveals the platinum resistance gene COX7B and the surrogate marker CD63. Cancer Med. 2018;7(12):6193‐6204. 30367559 10.1002/cam4.1828 PMC6308066

[44] Di X , Jin X , Ma H , et al. The oncogene IARS2 promotes non‐small cell lung cancer tumorigenesis by activating the AKT/MTOR pathway. Front Oncol. 2019;9:393. 31157169 10.3389/fonc.2019.00393 PMC6528107

[45] Fang Z , Wang X , Yan Q , Zhang S , Li Y . Knockdown of IARS2 suppressed growth of gastric cancer cells by regulating the phosphorylation of cell cycle‐related proteins. Mol Cell Biochem. 2018;443(1–2):93‐100. 29071539 10.1007/s11010-017-3213-8

[46] Lin H , Gao Y , Sun K , et al. COA3 overexpression promotes non‐small cell lung cancer metastasis by reprogramming glucose metabolism. Am J Cancer Res. 2022;12(8):3662‐3678. 36119836 PMC9442012

[47] Mamelak AJ , Kowalski J , Murphy K , et al. Downregulation of NDUFA1 and other oxidative phosphorylation‐related genes is a consistent feature of basal cell carcinoma. Exp Dermatol. 2005;14(5):336‐348. 15854127 10.1111/j.0906-6705.2005.00278.x

[48] Yang F , Hu A , Guo Y , et al. p113 isoform encoded by CUX1 circular RNA drives tumor progression via facilitating ZRF1/BRD4 transactivation. Mol Cancer. 2021;20(1):123. 34579723 10.1186/s12943-021-01421-8 PMC8474885

[49] Kou X , Ding H , Li L , Chao H . Caseinolytic protease P (CLPP) activated by ONC201 inhibits proliferation and promotes apoptosis in human epithelial ovarian cancer cells by inducing mitochondrial dysfunction. Ann Transl Med. 2021;9(18):1463. 34734015 10.21037/atm-21-4321 PMC8506775

[50] Su R , Dong L , Li Y , et al. Targeting FTO suppresses cancer stem cell maintenance and immune evasion. Cancer Cell. 2020;38(1):79‐96.e11. 32531268 10.1016/j.ccell.2020.04.017 PMC7363590

