# Supplementary figures and images for: A novel mitochondria‐related core gene signature to predict the prognosis and evaluate tumour microenvironment in CESC single‐cell validation
Source: J Cell Mol Med. 2024 Mar 27;28(8):e18265. doi: 10.1111/jcmm.18265 (PMC10967144; doi:10.1111/jcmm.18265)

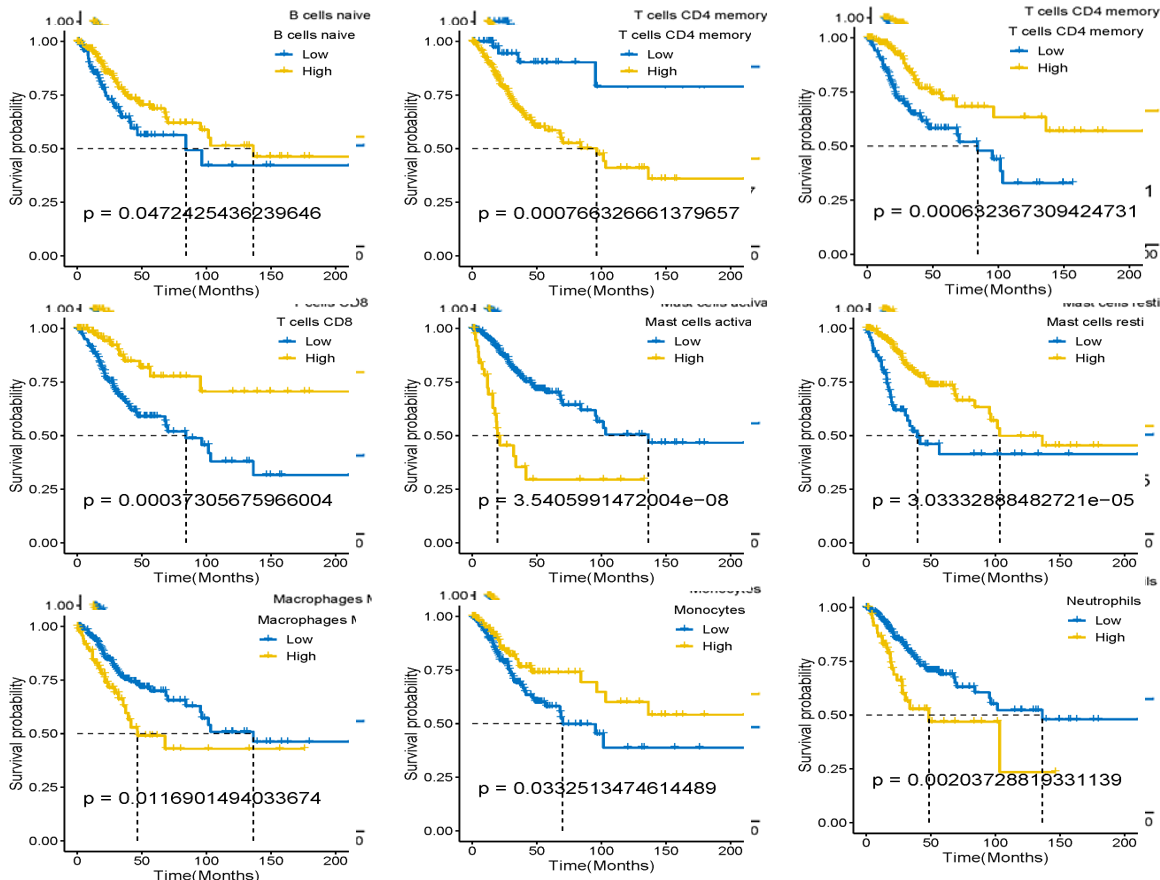

Supplement: Supplementary file 1 — Figure S1. [file JCMM-28-e18265-s002.png]

# Biological process enrichment

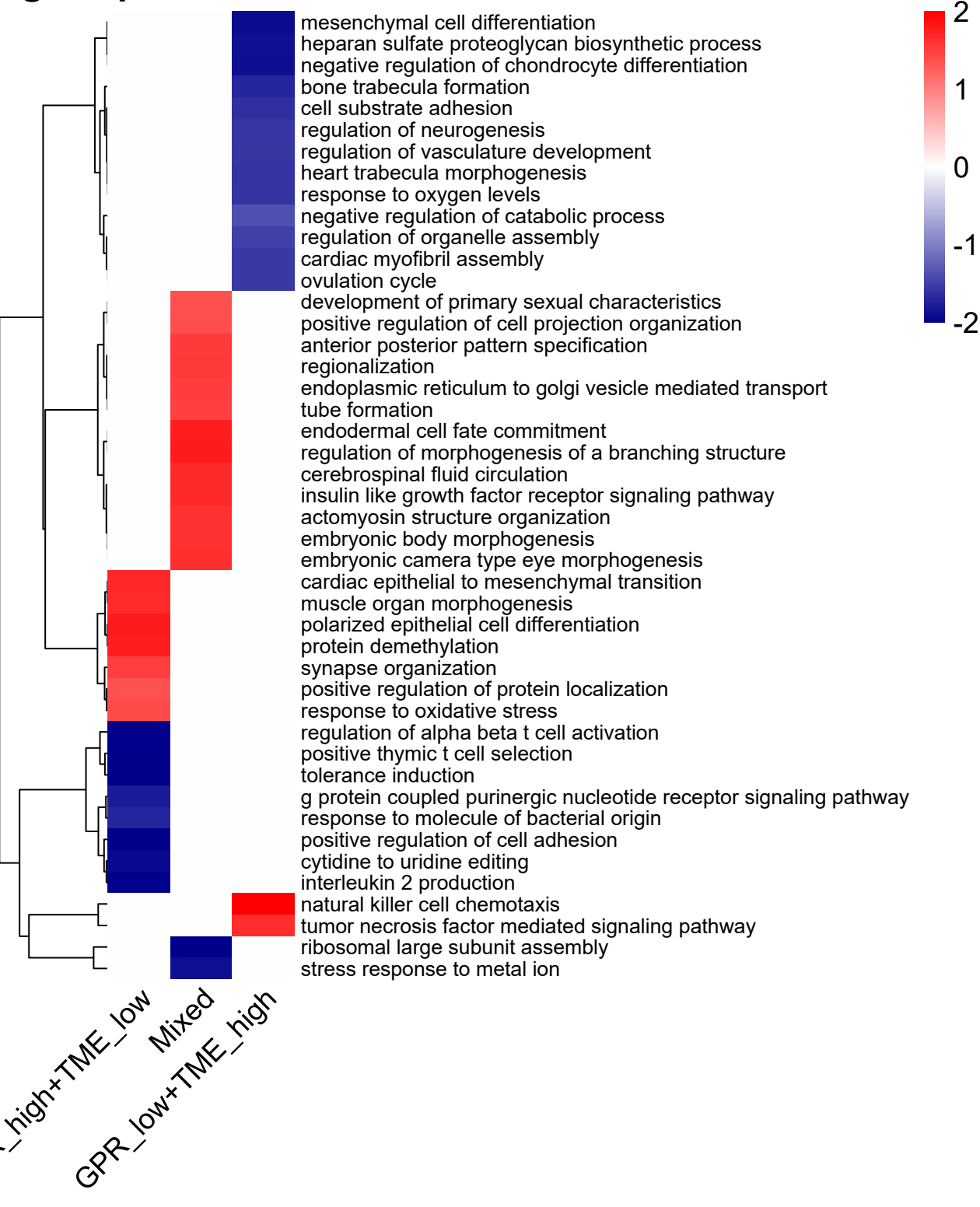

Supplement: Supplementary file 2 — Figure S2. [file JCMM-28-e18265-s001.pdf]
